# In silico assessment of electrophysiological neuronal recordings mediated by magnetoelectric nanoparticles

**DOI:** 10.1038/s41598-022-12303-4

**Published:** 2022-05-19

**Authors:** Ilhan Bok, Ido Haber, Xiaofei Qu, Aviad Hai

**Affiliations:** 1 Department of Biomedical Engineering, University of Wisconsin-Madison, Madison, WI USA; 2 Department of Electrical and Computer Engineering, University of Wisconsin-Madison, Madison, WI USA; 3 Department of Integrative Biology, University of Wisconsin-Madison, Madison, WI USA; 4 Grainger Institute for Engineering, University of Wisconsin-Madison, Madison, WI USA; 5 Wisconsin Institute for Translational Neuroengineering (WITNe), Madison, WI USA

**Keywords:** Biomedical engineering, Nanoparticles

## Abstract

Magnetoelectric materials hold untapped potential to revolutionize biomedical technologies. Sensing of biophysical processes in the brain is a particularly attractive application, with the prospect of using magnetoelectric nanoparticles (MENPs) as injectable agents for rapid brain-wide modulation and recording. Recent studies have demonstrated wireless brain stimulation in vivo using MENPs synthesized from cobalt ferrite (CFO) cores coated with piezoelectric barium titanate (BTO) shells. CFO–BTO core–shell MENPs have a relatively high magnetoelectric coefficient and have been proposed for direct magnetic particle imaging (MPI) of brain electrophysiology. However, the feasibility of acquiring such readouts has not been demonstrated or methodically quantified. Here we present the results of implementing a strain-based finite element magnetoelectric model of CFO–BTO core–shell MENPs and apply the model to quantify magnetization in response to neural electric fields. We use the model to determine optimal MENPs-mediated electrophysiological readouts both at the single neuron level and for MENPs diffusing in bulk neural tissue for in vivo scenarios. Our results lay the groundwork for MENP recording of electrophysiological signals and provide a broad analytical infrastructure to validate MENPs for biomedical applications.

## Introduction

Current whole-brain imaging technologies are either solely structural or provide some functional readouts that are limited in scope and indirect to electrophysiological signaling^1–4^. Relatively recent attempts at fully functional readouts mediated by injectable indicators include responsive molecular agents for magnetic resonance imaging (MRI)^5–9^ and functional ultrasound^10,11^, injectable microelectronic motes interacting wirelessly with noninvasive neuroimaging modalities^12–14^, and systemically expressed optogenetic constructs for whole-brain neural imaging in translucent animal preparations^15,16^. Magnetic particle imaging (MPI) is an emerging whole-body imaging modality exploiting the nonlinear magnetization of injected magnetic nanoparticles to achieve dynamic non-attenuated depth recordings with improved spatiotemporal resolution^17,18^. Recent studies demonstrate the use of MPI for brain applications including monitoring of neural injury^19^, tracking of brain graft cell migration^20^, assessing neuropathology requiring surgical interventions^21^, and several other functional characterizations of cerebral blood volume during brain activation^22–25^. The majority of MPI studies rely on the injection of superparamagnetic iron oxide nanoparticles (SPIONs) to acquire concentration-dependent readouts of diffused particles. New magnetic particle designs that offer signal modulation specific to biochemical and physiological processes can create a new repertoire of readouts for MPI.

Developments in the synthesis of magnetoelectric nanoparticles (MENPs) and related structures^26–28^ have given rise to diverse material traits that could empower MPI with dynamic readouts relevant to physiology and neurophysiology. Recent research on the magnetoelectric effect has predominantly focused on the characterization of new material substrates^29–31^ and the simulation of lattice interfacial coupling^32,33^. Finite element modeling (FEM) solvers in particular are used to better characterize a diverse range of magnetoelectric geometric arrangements and structures^34–36^. MENPs and similar heterostructures can be externally modulated by electric and magnetic fields, and have been successfully applied for applications including neurostimulation^37,38^, neural recording^39,40^, tumor ablation^41,42^, drug delivery^41–43^, and magnetically controlled nanorobots^44^. These studies and further demonstrations of biological compatibility^45^ establish MENPs as injectable agents for in vivo preparations allowing for both acute and chronic studies. Further development on magnetoelectric transistors^46^, biocompatible implantable devices^47^, and integrated brain-computer interfaces^39,48^ could serve as powerful new platforms for studying and managing a wide set of pathologies.

One of the most common composites used in these efforts is a cobalt ferrite (CFO) and barium titanate (BTO) core–shell conjugate (CFO–BTO) due to its high magnetoelectric coupling coefficient and relatively low toxicity, but other emerging composites such as BTO/iron oxide^49,50^ (*α* = 28.78 mV/cm·Oe), cobalt-doped BiFeO_3_^51,52^ (*α* = 6.5 V/cm·Oe), CFO/BTO/polydopamine-P(VDF-TrFE)^53^ (*α*_*E33*_ = 150.58 mV/cm·Oe), and BTO/nickel^54^ (*α* = 225 µV/cm·Oe) are paving the way to an expanded toolkit of magnetoelectric probes and sensors. With appropriate biocompatible surface functionalization, new magnetoelectric compounds are expanding usability and improving safety for both SPIONs and MENPs for MPI^55–57^. Histological analysis of injected CFO–BTO MENPs in mice demonstrates long-term degradation and excretion^45^, and additionally, administration across the blood–brain barrier has been shown by way of intranasal injection in mice^58^. These findings lead to proposing MENPs for use in conjunction with MPI towards enabling direct volumetric readouts of neurophysiological events^59^, presenting estimations of MPI signal change in response to macro-scale electric fields in the brain. However, a computational framework that quantifies particle-level magnetostrictive modulation of CFO–BTO MENPs by neuronal electric fields and combines it with realistic cell morphologies, spiking activity, and particle diffusion has not been developed yet. This has precluded proper determination of the conditions whereby MENPs can be used to acquire direct electrophysiological recordings for experimental implementation.

This study lays the theoretical groundwork for using MENPs to detect neuronal electric fields based on the nonlinear magnetization effect exploited in MPI. We first establish a finite element nanoscale model for CFO–BTO MENPs and quantify their modulation by oscillating external fields. We then simulate magnetization modulation by nearby physiologically-relevant electric fields, and optimize the core–shell ratio for maximal responsiveness and to inform synthesis for sensitive MENPs. Finally, we apply the model to different neuronal morphologies and quantify magnetic field strength across cellular compartments during action potentials at a given MENP concentration and diffusion rate in the brain. This work presents a realistic quantification of the expected MPI signal change using MENPs as the agents injected into neural tissue. More broadly, our model offers a framework that can be applied to assess MENPs for versatile sensing applications.

## Results

### Nonlinear magnetization properties of SPIONs and MENPs

**Figure 1:**
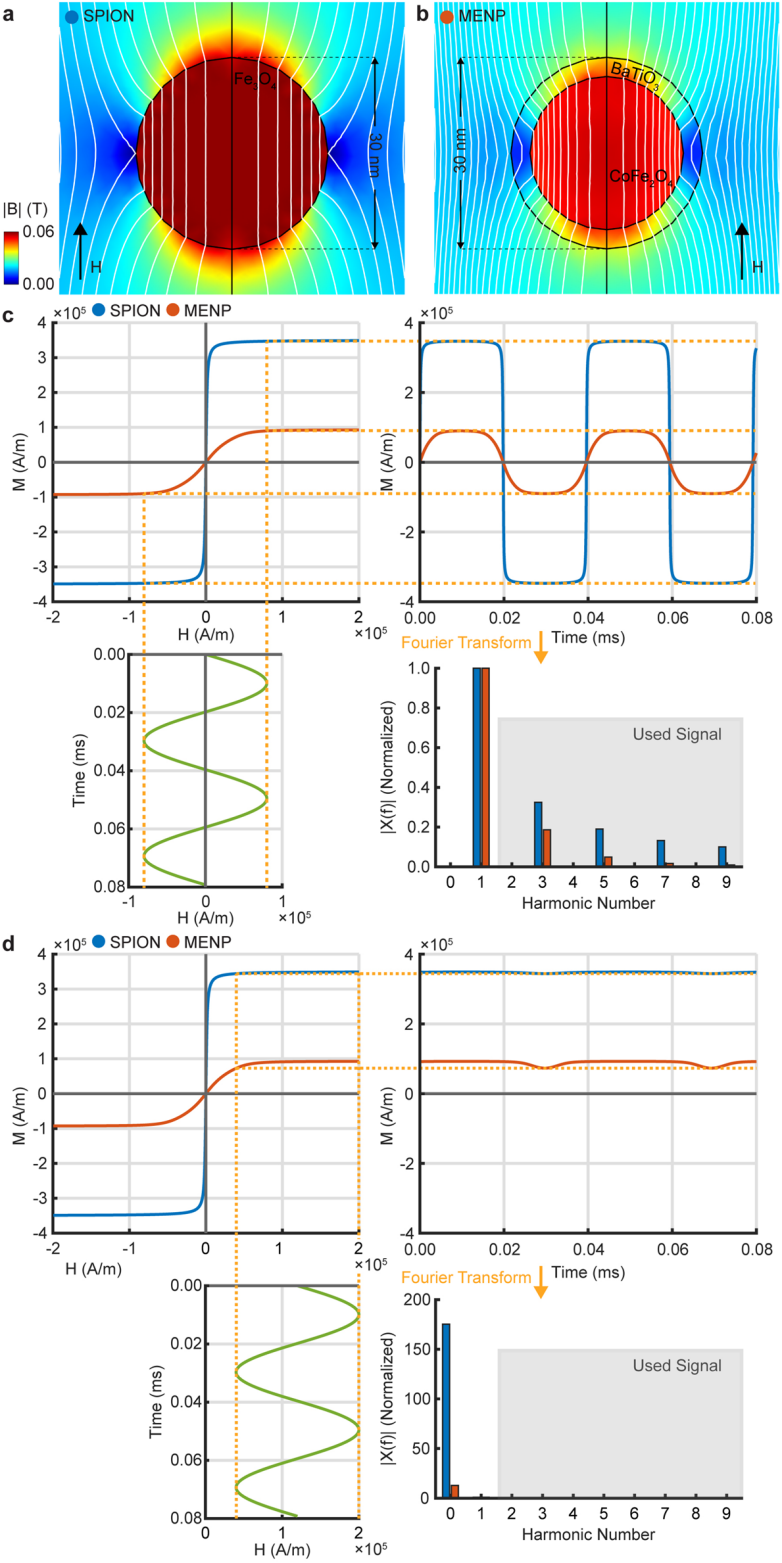
Magnetic flux density and magnetization harmonics for SPIONs and MENPs. (**a**) Magnetic flux density amplitude of a 30 nm SPION in response to 40 kA/m *H-*field (white contour lines—magnetic flux lines). (**b**) Response of a 30 nm CFO–BTO MENP to the same field. (**c**) Nonlinear magnetization and Fourier transform harmonics for SPIONs and MENPs in response to a 25.25 kHz, 80 kA/m oscillating *H-*field. (**d**) Magnetization saturation for SPIONs and MENPs with a 120 kA/m bias field to a 25.25 kHz, 80 kA/m oscillating *H-*field. For (**c**) and (**d**) values are normalized to the first harmonic of each particle type.

We began by validating our model for SPIONs (Fig. 1a,c,d, blue) compared with known nonlinear magnetization properties used in MPI^17,18^. Nanoparticles of diameter *d* = 30 nm experienced a magnetic field of *H* = 40 kA/m resulting in a dipole with a maximal absolute magnetic flux density of 62.3 mT across the applied field (Fig. 1a). For an alternating *H-*field, SPIONs displayed nonlinear magnetization saturation at ± 347.1 kA/m (Fig. 1c,d, green: alternating *H*-field, blue: SPION magnetization) consistent with reported values^60,61^. We next evaluated the response of CFO–BTO MENPs under the same conditions (Fig. 1b–d, red). The maximal absolute magnetic flux density for MENPs was 58.4 mT (Fig. 1b) and nonlinear magnetization saturation in response to alternative fields was observed at ± 89.9 kA/m (Fig. 1c,d, red: MENPs magnetization). We quantified signal harmonics used for signal detection with *H*-field alternating between ± 80.0 kA/m at a frequency of 25.25 kHz applied to both SPIONs or MENPs (Fig. 1c, bottom right, red and blue, respectively), with values normalized to the first harmonic. MENPs displayed odd harmonics amplitude ratios comparable to SPIONs, with 100%, 15.80%, 3.54%, and 1.36% for first, third, fifth, and seventh harmonics, respectively, for MENPs. This is compared with 100%, 32.48%, 18.98%, and 13.20% for first, third, fifth, and seventh harmonics, respectively, for SPIONs. The addition of a bias field to the oscillating *H-*field resulted in negligible harmonics for both MENPs and SPIONs that were magnetically saturated. The presence of MENPs can thus be detected at normal MPI settings using odd harmonics of nonlinear magnetization despite differences in magnetic flux density distribution.

### Effect of core size on magnetization modulation in magnetoelectric nanoparticles

**Figure 2:**
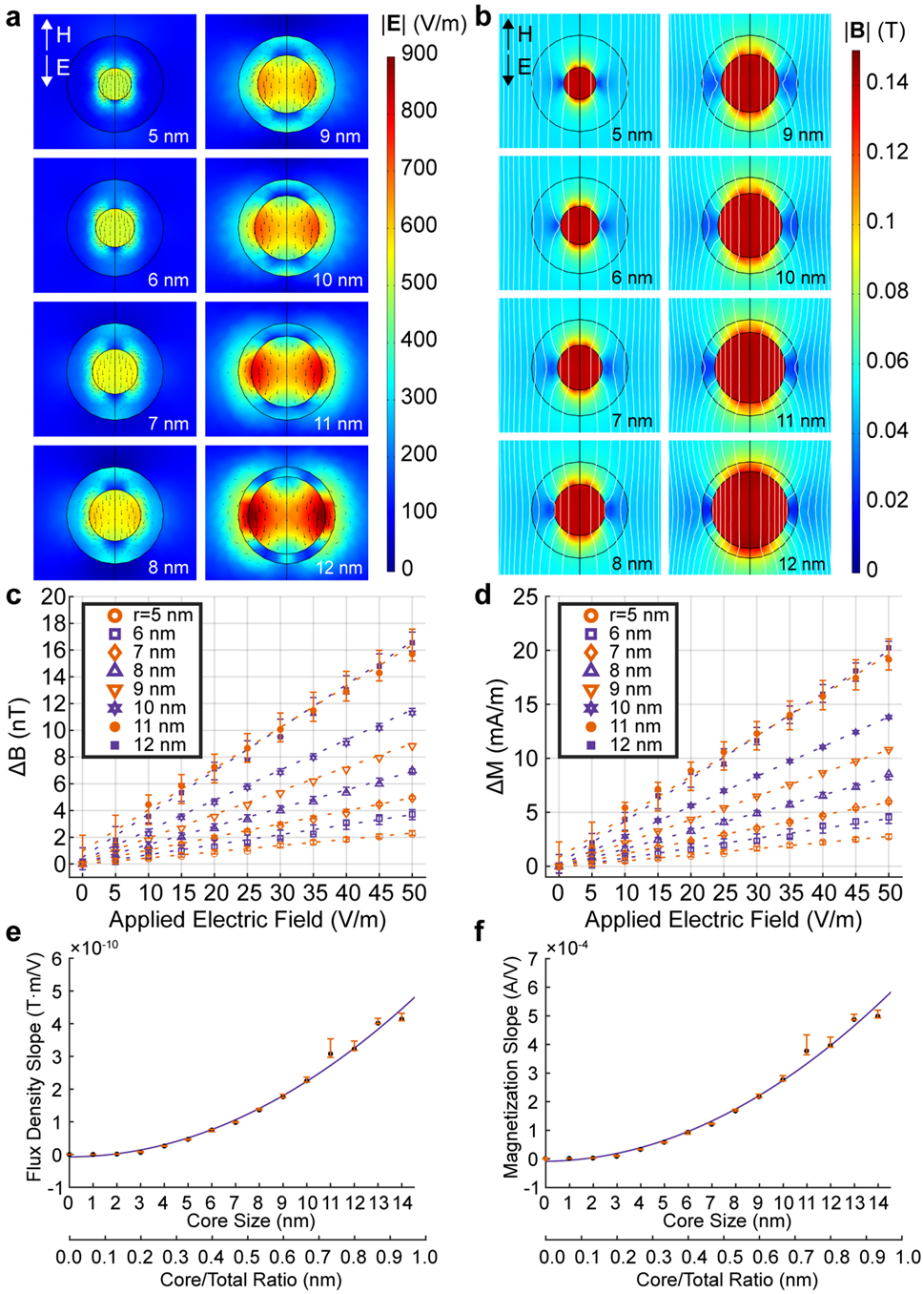
Effect of MENP core size on electric field-based magnetization modulation. (**a**) The effect of core size on electric field magnitude (colormap) and direction (vectors) in response to external fields. The core radius ranged from 5 to 12 nm corresponding to shell thickness ranging from 10 to 3 nm. In all cases, E_z_ = − 100 mV/mm antiparallel to H_z_ = 4 kA/m. (**b**) Magnetic flux density plots for the same configurations in (**a**). (**c**) Volume-averaged changes in magnetic flux in response to electric field for the same core sizes. (**d**) Volume-averaged changes in magnetization in response to electric field, across the same core sizes. (**e**) Slope of the magnetic flux modulation versus electric field linear slope. (**f**) Slope of the magnetization modulation versus electric field linear slope. For both (**e**) and (**f**), the abscissa is labeled with both core size and core/total ratio. Error bars denote 95% confidence intervals for all panels.

Previous simulations^36^ and synthesis^62^ of CFO–BTO MENPs with increasing core–shell ratios were shown to directly affect magnetoelectric coupling and can be leveraged to optimize the sensitivity of MENPs to neuronal electric fields. We evaluated the relationship between CFO core size and magnetic flux density amplitude in the presence of physiologically relevant electric fields ranging between 0 and 50 mV/mm (Fig. 2). An electric field opposing a 4 kA/m *H-*field was applied to 30 nm MENPs with CFO core radii ranging between 5 and 12 nm corresponding to BTO shell thicknesses ranging between 10 and 3 nm (Fig. 2a,b, see also Fig. S1). Overall core–shell average magnetic flux density at 50 mV/mm increased with larger core sizes and ranged between 5.356 mT (5 nm) and 9.629 mT (12 nm) under the same electric field and antiparallel 4.0 kA/m *H-*field configuration (Fig. 2b). Average magnetic flux density in the core at 50 mV/mm remained relatively constant at between 13.92 mT (multiple sizes) and 14.08 mT (12 nm) independent of core size and consistent with the high permeability of CFO relative to BTO. We then quantified the magnetic flux density (Fig. 2c) and corresponding magnetization (Fig. 2d) for different core sizes in response to different electric fields ranging between 0 and 50 mV/mm. We find a nonlinear increase in sensitivity to electric fields reaching 0.414 nT m/V and 0.497 mA/V for 30 nm CFO–BTO MENPs with 14 nm CFO core radius (Fig. 2e,f). Our findings correlate with similar magnetoelectric structures characterized elsewhere^63–74^ and demonstrate comparable shell displacement (Fig. S2)^75^, affirming that further optimization will require a large core–shell ratio.

### Field directionality-dependent magnetization amplitude

**Figure 3:**
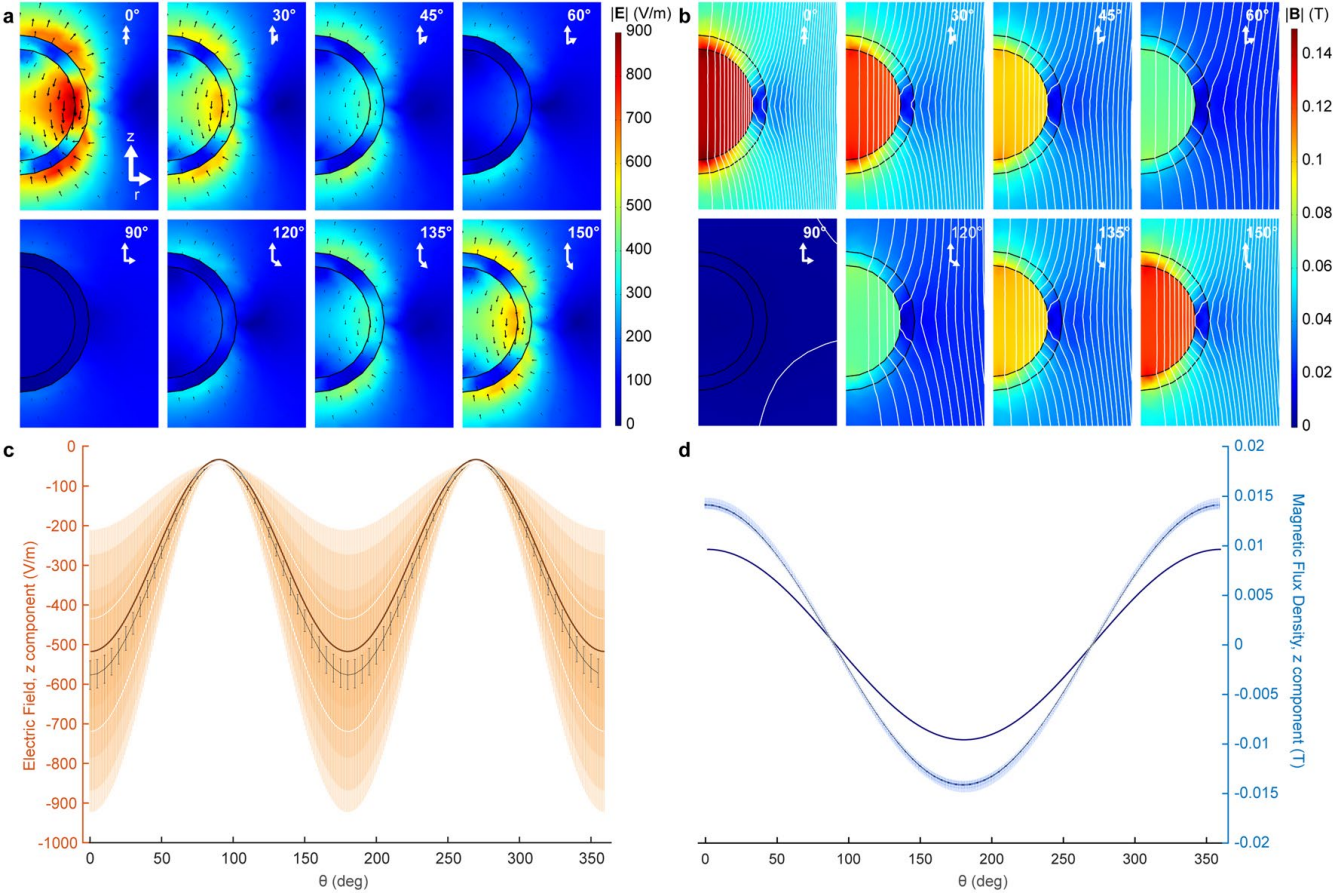
Effect of applied electric field and magnetic field intensity directionality on measured MENP electric field and magnetic flux density. (**a**) Electric field norm and vector plots for selected angles (0°, 30°, 45°, 60°, 90°, 120°, 135°, 150°). (**b**) Magnetic flux density norm and contour plots for the same angles in (**a**). (**c**) Mean electric field z component (thick brown trace), interquartile range (thin white traces) and decile plot (shades of orange) relative to the angle between applied electric field and *H-*field. Deciles plotted are (0–100), (10–90), (20–80), (30–70), (40–60), and the mean (50—central black trace). Deciles closest to the mean are not visible due to their low range. (**d**) A magnetic flux density plot for the same conditions as in (**c**); shown are mean magnetic flux density z component (navy blue), interquartile range (white), and deciles (shades of blue). Black error bars denote standard error of the mean for all panels.

MPI tomography relies on injected magnetic nanoparticles experiencing an externally applied *H-*field. Directionality of the external field applied on MENPs relative to in situ electric fields of diverse neuronal morphologies and orientations is expected to affect detectability. We explored this effect by modifying the angle *θ* between the electric field and *H-*field for MENPs (Fig. 3). For both electric field (Fig. 3a) and magnetic flux density (Fig. 3b) we find the maximum average effect at 0° and 180° of 539.82 V/m and 10.14 mT, respectively, minimized at 90° and 270° with values decreasing to 35.62 V/m and 0.13 mT (see Fig. S3 for corresponding magnetization plots and diagrams, and Movies S1–Movies S3 for a 360° sweep of all three parameters). Average electric field and magnetic flux density across the particle (Fig. 3c) and decile plots (Fig. 3d) demonstrate that 50% of the magnetization occurs at 22.2% of the total volume of the particle at optimal angles, with 99.942% occurring at the core and 0.058% occurring at the shell. Our directionality estimates allow for proper quantification of the expected MPI signal recorded in response to electric fields generated by excitable cells with diverse compartmental anatomy in the presence of MENPs.

### Quantification of MENP magnetization response from single neurons

**Figure 4:**
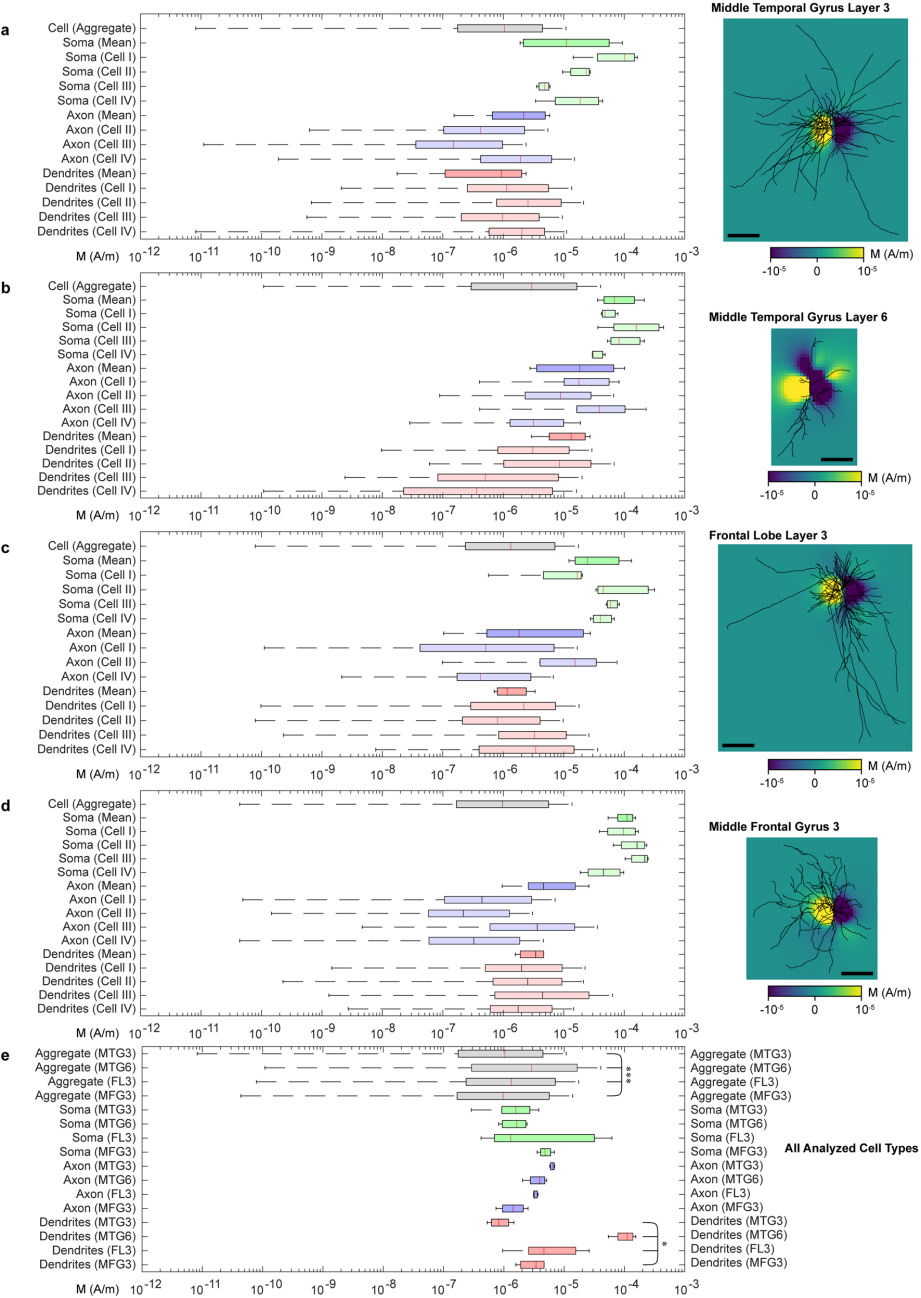
Distribution of magnetization from single spiking neurons. Shown above are magnetization maps of a spiking cortical layer 3 middle temporal gyrus neuron (**a**), layer 6 middle temporal gyrus neuron (**b**), layer 3 frontal lobe neuron (**c**), and a layer 3 middle frontal gyrus neuron (**d**). Shown in right panels in (**a**)–(**d**) are magnetization magnitude and sign for a bias field in the x direction. The slices are taken from the x–z plane. The left panels show the absolute value magnetization mean (red line), 1st and 3rd quartiles (colored boxes), and outliers (Q1 -1.5*IQR; Q3 + 1.5*IQR) for all regions of all cells, as well as for total aggregated data, on logarithmic scale. Scale bar = 200 µm for (**a**–**d**). (**e**) Salient bar and whisker entries from cell types in panels a, b, c, and d. Note the statistical significance (*p < 0.05, ***p < 0.001, one-way ANOVA) between dendritic compartments and total cell aggregate data.

CFO–BTO MENPs in concentrations ranging between 50 and 200 µg/mL were demonstrated to be compatible with in vivo brain applications^37,38,43^. We turned to quantifying the expected magnetic field strength arising from the excitation of single neurons for sensing activity in the presence of CFO–BTO MENPs at comparable concentrations (Fig. 4). Maximal absolute magnetization during action potential peak integrated over the morphological volume was between 8.228 × 10^–12^ and 9.635 × 10^–3^ A/m surrounding neuronal somata, axons, and neurites of multiple cortical morphology types (layer 3, middle temporal gyrus; layer 6, middle temporal gyrus; layer 3, frontal lobe; layer 3, middle Frontal gyrus; Fig. 4a–d, n = 4 for each type) and varied significantly between all types (F = 24.5, p = 7.90429 × 10^–16^; one-way ANOVA, Fig. 4e). MENP concentration was 117.5 µM corresponding to 1415 particles/µm^2^. Magnetization proximal (*r* = 20 µm) to somatic in silico compartments ranged between 1.901 × 10^–6^ and 2.147 × 10^–4^ A/m (absolute value), varying insignificantly between cell types (F = 1.73, p = 0.21473; one-way ANOVA). Absolute magnetization proximal to axons ranged between 1.033 × 10^–7^ and 1.016 × 10^–4^ A/m, also varying insignificantly between cell types (F = 1.06, p = 0.40931; one-way ANOVA). Absolute magnetization arising from dendritic trees, however, ranged between 1.749 × 10^–8^ and 2.714 × 10^–5^ A/m and varied significantly between cell types (MTG3, MTG6, FL3, MFG3; F = 5.11, p = 0.01655; one-way ANOVA). This magnetization corresponds maximally to 3.41 nM iron (20 nM Fe gives 5% of 4 × 10^–9^ T/μ_0_—the magnetization of a proton in a 1 T MRI field) and thus detectable fields by MPI^17^ and MEG^76^. Significant differences between cell types and subcellular dendritic compartments indicate the ability to differentiate between cell types and brain regions by MENP magnetization amplitude.

### Monte Carlo simulations of diffusing MENPs in interconnected neuronal networks

**Figure 5:**
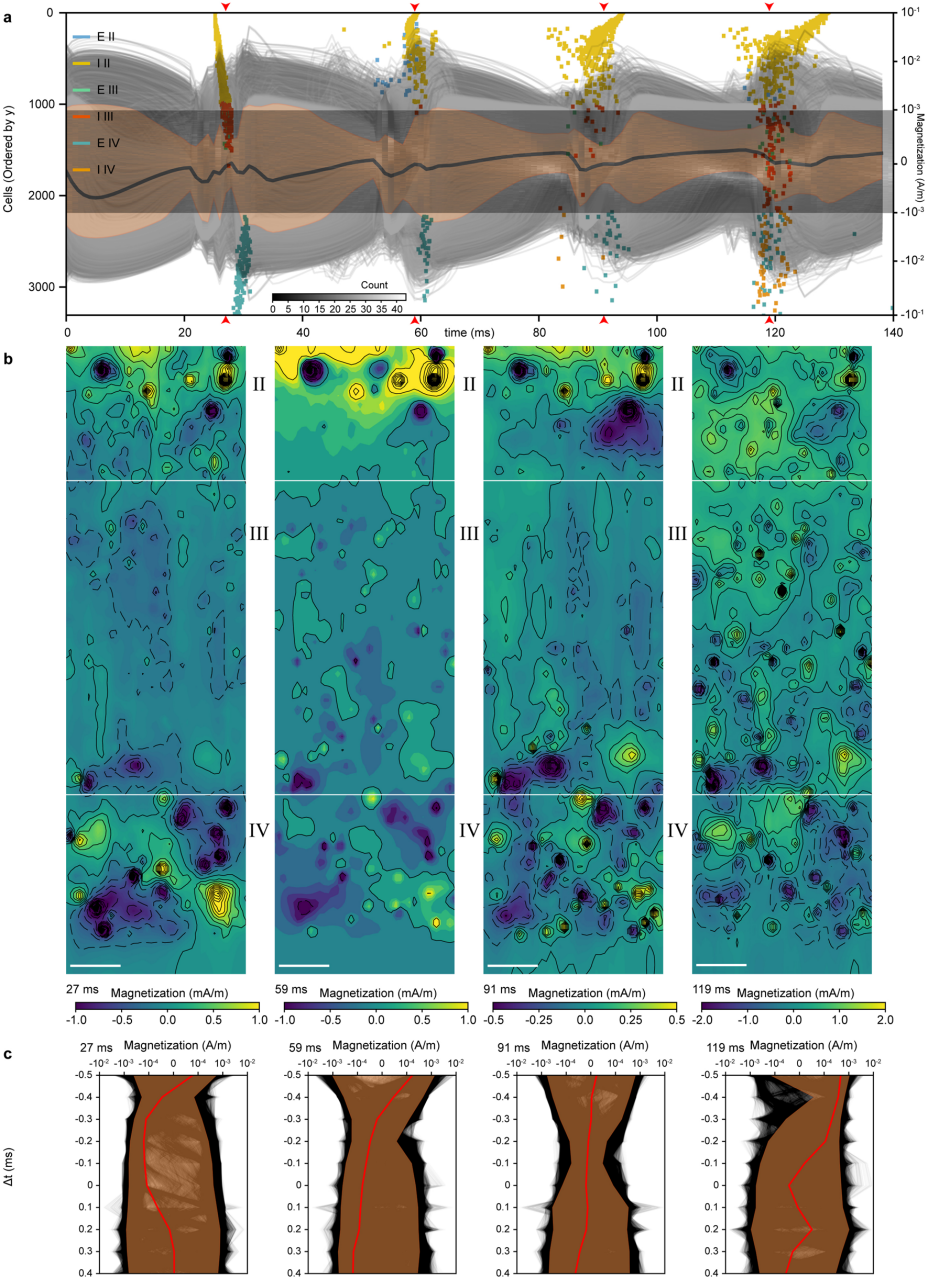
Magnetization of MENPs within a simulated cortical voxel. (**a**) Firing activity of neurons within the simulated neocortical slice (E = excitatory, I = inhibitory cells) over a 140 ms period. Layers II, III, and IV of the neocortex were simulated, each 150 µm, 350 µm, and 200 µm thick, respectively, (total thickness = 700 µm). Mean firing rates were 6.20 Hz and reached 19.13 Hz during network bursts. Composite magnetization traces (grayscale) overlaid on a time-dependent histogram (grayscale). Grayscale traces are the magnetization at recording sites color-coded by distance from the slice center (lighter = further from center). The thick black line is the mean magnetization in the slice, while the orange swath is the standard deviation. The time-dependent histogram covers the linear regime of the symmetric log plot (from −1 to 1 mA/m), with bin dimensions of 1 ms by 5 μA/m. (**b**) Static yz magnetization colormaps through x = 100 µm for each of four timepoints marked by red arrows in panel a). Scale bar = 50 µm. (**c**) Monte Carlo magnetization simulations for perfused MENPs. Single particles were centered at each of the four times (i.e. 27 ms, 59 ms, 91 ms, 119 ms) and traversed through the neural network vertically from the top (y = 0 µm) to bottom (y = 700 µm) edge. The thick red line is the mean magnetization, and the orange swath is the standard deviation.

To gain a realistic assessment of response to multicellular neural activity in vivo, we quantified magnetization of MENPs at a concentration of 27.5 μg/mL within a 700 µm deep cortical section comprising Layers II, III, and IV (150 µm, 350 µm, and 200 µm deep, respectively) and a total of 237,021 extracellular recording sites interspaced by 5 µm (Fig. 5). The network included excitatory and inhibitory cells similar to reported ratios^77,78^ spiking at overall frequencies of 5.30 Hz and 6.34 Hz and up to 16.77 Hz and 19.50 Hz during network bursts, respectively (Fig. 5a). Vectorized extracellular potentials served as inputs to the MENP directionality matrix (Fig. 4) for each recording site, enabling calculation of the magnetization at different coordinates within the cortical voxel over a 140 ms period (Fig. 5a, grayscales traces, magnetization at 237,021 recording sites). The mean signal arising from the network reached maximal amplitudes of 1354.34 µA/m (Fig. 5a, black trace) and mean amplitude of 219.8138 µA/m to 381.1203 µA/m during network bursts (Fig. 5a, arrows, and Fig. 5b, four magnetization maps across a cortical slice corresponding to *t* = 27 ms, 59 ms, 91 ms, and 119 ms).

Nanoparticles injected intravenously travel through vasculature at speeds of 65 ± 12 cm/s^79^, with a diffusion coefficient of 48–15 µm^2^/s for sizes ranging from 10.4 to 32.0 nm^80,81^. We evaluated the signal arising from MENPs perfused through a cortical voxel at 650 µm/ms with a series of 40,000 Monte Carlo simulations of MENPs at the four network bursts originating from random coordinates in the x–z plane (y = 0) and propagating along the y axis over a 1.0 ms period centered at network burst peaks (Fig. 5c). The MENP signal arising from network activity with perfused MENPs reached a mean amplitude of 59.3402 to 417.6602 µA/m during network bursts (Fig. 5c). The maximum collective signal during network bursts arising from static MENPs relevant to particles penetrating through the blood–brain barrier^45^ and settling in the brain interstitium was 36.5399 µA/m lower compared with perfused MENPs traveling through the vasculature and forms a more realistic estimation of using MENP-mediated MPI to record electrophysiological events.

## Discussion

This study provides a realistic platform for quantifying the magnetization of magnetoelectric nanoparticles (MENPs) for sensing neurophysiological electric fields with cellular-level precision. We established a finite element strain-based model that emulates piezoelectric deformation of a BTO shell over a CFO nanoparticle core in response to small extracellular electric fields, giving rise to magnetic flux detectable by low-field modalities^82,83^ and suitable for brain recording. CFO–BTO MENPs modeled here are emerging as agents for brain applications^37,38,59^ and are shown to traverse the blood–brain barrier with minimal adverse effects^45^. Other core–shell combinations are also being developed and offer increased magnetoelectric coupling with comparable biocompatibility^50,53,54^. Our model can be generalized for quantifying such diverse compounds by integration with more advanced time-domain equations^84,85^ and serve as a comprehensive tool for characterizing magnetoelectric materials and for quantifying sensitivity to biophysical phenomena in multiple systems.

Patterned magnetoelectric stacks^70,86^, nanowires^87^, matrices^88,89^, and heterostructures^65,90–92^ are of particular interest and were introduced as more versatile and scalable platforms responsive to electric fields. These can serve as multiplexed arrays for spatially precise readouts and stimulation of neural activity, and integrate with other magnetoelectric technologies for brain recording^39,40,93^ and stimulation^37,48,94^. Finite element three-dimensional analyses can be specifically leveraged to characterize diverse device geometries in addition to simple core–shell particles shown here and can be used for optimized sensing of biogenic electric fields.

Our particle model predicts magnetization of MENPs at a physiological concentration of 117.5 µM (27.495 μg/mL) administered extracellularly to excitable neurons with diverse morphologies. We predict an 8.228 × 10^–12^ to 9.635 × 10^–3^ A/m response from single neurons at peak membrane depolarization, and 59.3402–417.6602 µA/m across a 200 × 700 × 200 µm^3^ voxel for multicellular interconnected networks of neurons mimicking in vivo scenarios. Sensitivity of detection and spatiotemporal resolution in MPI depend on nanoparticle size^95^. A concentration of 5 mg/mL for nanoparticle diameters of 18.5 nm to 32.1 nm^95–99^ corresponds to a FWHM of 16.7 mT/µ_0_ to 25.1 mT/μ_0_, respectively at 20.25 kHz with a 20 mT input sinusoid, for single volumetric acquisitions. Modulation of 10 mA/m in MENPs is equivalent to a concentration difference of 0.933 ng/mL for simple SPIONs detectable in MPI^99,100^. Our results suggest sufficient sensitivity to extracellular electric fields assuming MENP concentrations greater than 117.5 µM (27.495 μg/mL) for stationary MENPs and intravenously perfused MENPs. MENPs localized directly on the plasma membrane can increase detectability further assuming particles experience electric fields that correspond to full intracellular membrane potential differences^59^. Wang et al. report 0.3 emu/g saturation for MENPs (1.8 kA/m assuming the particle has a density close to CFO of 6.02 g/cc^101^) for an immobilized single-layer array^102^ and Etier et al. report 20 emu/g (120.4 kA/m assuming the same) for both a loose powder and fixed powder. Etier et al. note that hysteresis is not present in the loose powder, because the particles can freely rotate^103^. This is as seen clinically in vivo, which our model represents. The temporal resolution for single point recordings used in MPI spectroscopy reaches sub-millisecond scales^104^ and exhibits negligible hysteresis at neuronal time scales^105^, allowing for improved temporal sensitivity even to single field potential events.

Existing particle-based neuroimaging systems for neurochemical and neurophysiological readouts require hundreds of milliseconds for single acquisitions^8,106^ and can be enhanced by the high temporal resolution of MENP-based MPI technology. Voltage-sensitive MENPs can also increase coverage in the brain and supplement voltage-sensitive optical dyes currently used as injectable or genetically expressed agents for cell-type-specific readouts^107^, in addition to established optogenetic tools for neural stimulation^108^. MENPs are capable of bidirectional brain recording and stimulation and can thus serve as magnetoelectric equivalents of optical tools, enabling greatly increased recording depth and signal penetration.

## Conclusion

Magnetoelectric materials are increasingly used for biomedical sensing and modulation, and provide minimally invasive access to different organ systems and the brain in particular. In this study, we describe an in silico characterization framework to assess the response of cobalt ferrite (CFO) barium titanate (BTO) core–shell magnetoelectric nanoparticles (MENPs) to neural electric fields and investigate feasibility for wireless electrophysiological readouts using injectable magnetoelectric agents. The magnitude of magnetoelectric coupling from different core–shell ratios is analyzed and optimized, and the direction-dependent electric and magnetic field distributions are presented. The resulting time-dependent magnetoelectric responses from single neuronal morphologies and realistic neural networks during MENP perfusion were statistically quantified, and the induced magnetic fields were found to be within the detectability limits of magnetic particle imaging (MPI). Our model is applicable to numerous other geometries and material configurations, enabling the validation of other potential magnetoelectric transducer designs and the advent of novel applications of magnetoelectric materials in biomedicine.

## Methods

### Nanoparticle modeling

A strain-based finite element model was applied to simulate the magnetoelectric effect for CFO–BTO and SPIO nanoparticles using custom equations in COMSOL Multiphysics 5.6 (COMSOL Inc. Stockholm, Sweden). The Langevin equation was employed to derive the M–H curve for the SPION model^109^. The M–H relationship for BTO was acquired by tracing an M–H curve based on previous studies^110^. For CFO–BTO, two concentric spheres were formed, the inner sphere representing magnetostrictive CFO and the outer shell representing piezoelectric BTO. The *Electrostatics*, *Magnetic Fields*, and *Solid Mechanics* modules were used, along with *Magnetostriction* and *Piezoelectric Effect* multiphysics couplings. Electrostatic modeling of non-piezoelectric media used the *Charge Conservation* boundary condition and was based on Gauss’s Law:

1 $$\nabla \cdot \overrightarrow{{\varvec{D}}}= {\rho }_{v}$$

where $\nabla$ is the del operator, ***D*** is the electric flux density and *ρ*_*v*_ is the volume charge density. Modeling with piezoelectric media used linear piezoelectric coupling (boundary condition: *Charge Conservation, Piezoelectric*):

2 $$\nabla \cdot \left({\epsilon }_{0}\overrightarrow{{\varvec{E}}}+{\epsilon }_{0}{\chi }_{rs}\overrightarrow{{\varvec{E}}}+{\varvec{e}}:{\varvec{\varepsilon}}\right)= {\rho }_{v}$$

where ${\epsilon }_{0}$ is the electric permittivity, ***E*** is the electric field intensity, *χ*_*rs*_ is the relative electrical susceptibility, ***e*** is the piezoelectric Voigt coupling matrix representing the stress tensor, and ***ε*** is the strain tensor. Magnetic field modeling was performed based on Ampere’s Law:

3 $$\overrightarrow{{\varvec{B}}}=\nabla \times \overrightarrow{{\varvec{A}}}$$

and

4 $$\nabla \times \overrightarrow{{\varvec{H}}}=\overrightarrow{{\varvec{J}}}$$

5 $$\overrightarrow{{\varvec{J}}}=\sigma \overrightarrow{{\varvec{E}}}+\sigma \overrightarrow{{\varvec{v}}}\times \overrightarrow{{\varvec{B}}}+\overrightarrow{{{\varvec{J}}}_{{\varvec{e}}}}$$

where ***B*** is the magnetic flux density, ***A*** is the magnetic vector potential, ***H*** is the magnetic field intensity, ***J*** is the electric volumetric current density, *σ* is the electrical conductivity, ***v*** is net charge velocity, and ***J***_***e***_ is electron current density. The constitutive relation between magnetic flux density, magnetic field intensity, and magnetization varied by domain. For cerebrospinal fluid (boundary condition: *Ampere’s Law*),

6 $$\overrightarrow{{\varvec{B}}}=\mu \overrightarrow{{\varvec{H}}}$$

the BTO shell (boundary condition: *Ampere’s Law*),

7 $$\overrightarrow{{\varvec{B}}}={\mu }_{0} (\overrightarrow{{\varvec{H}}}+\overrightarrow{{\varvec{M}}})$$

and for the CFO core (boundary condition: *Ampere’s Law, Magnetostrictive*),

8 $$\overrightarrow{{\varvec{B}}}={\mu }_{0} (\overrightarrow{{\varvec{H}}}+\overrightarrow{{\varvec{M}}}\left(\overrightarrow{{\varvec{H}}},{\varvec{S}}\right))$$

respectively, where *μ* is the magnetic permeability, ***M*** is the magnetization, and ***S*** is the stress tensor (see Eqs. (12) and (14)). For modeling linear elastic media (boundary condition: *Linear Elastic Material*),

9 $$0=\nabla \cdot {\varvec{S}}+{{\varvec{F}}}_{{\varvec{v}}}$$

10 $${\varvec{\varepsilon}}=\frac{1}{2} \left[{\left(\nabla \overrightarrow{{\varvec{u}}}\right)}^{T}+\nabla \overrightarrow{{\varvec{u}}} \right]$$

where ***F***_***v***_ is the volume deformation tensor and ***u*** is the solid displacement vector. Piezoelectric stress was modeled by (boundary condition: *Piezoelectric Material*)

11 $${\varvec{S}}={S}_{0}+\overrightarrow{{\varvec{C}}}:{\varvec{\varepsilon}}-\overrightarrow{{\varvec{E}}}\cdot {\varvec{e}}$$

(where *S*_*0*_ is the initial stress, and ***C*** is the elastic right Cauchy deformation tensor) and for magnetostrictive stress (boundary condition: *Magnetostrictive Material*)

12 $${\varvec{S}}={S}_{0}+{{\varvec{c}}}_{{\varvec{H}}}:\left[{\varvec{\varepsilon}}-{{\varvec{\varepsilon}}}_{{\varvec{m}}{\varvec{e}}}\left(\overrightarrow{{\varvec{M}}}\right) \right]$$

where ***c***_***H***_ is the elasticity tensor, and ***ε***_***me***_ is the magnetostrictive strain,

13 $${{\varvec{\varepsilon}}}_{{\varvec{m}}{\varvec{e}}}=\frac{3}{2}\frac{{\lambda }_{s}}{{M}_{s}^{2}}dev (\overrightarrow{{\varvec{M}}}\otimes \overrightarrow{{\varvec{M}}})$$

where *λ*_*s*_ is the saturation magnetostriction, and *M*_*s*_ is the saturation magnetization, matching the behavior of CFO nanoparticles in dispersion. Furthermore,

14 $$\overrightarrow{{\varvec{M}}}={M}_{s}L (\left|{\overrightarrow{{\varvec{H}}}}_{{\varvec{e}}{\varvec{f}}{\varvec{f}}}\right|)\frac{{\overrightarrow{{\varvec{H}}}}_{{\varvec{e}}{\varvec{f}}{\varvec{f}}}}{|{\overrightarrow{{\varvec{H}}}}_{{\varvec{e}}{\varvec{f}}{\varvec{f}}}|}$$

where *L* is the Langevin function, and

15 $${\overrightarrow{{\varvec{H}}}}_{{\varvec{e}}{\varvec{f}}{\varvec{f}}}=\overrightarrow{{\varvec{H}}}+\frac{3{\lambda }_{s}}{{\mu }_{0}{M}_{s}^{2}}dev\left({\varvec{S}}\right)\overrightarrow{{\varvec{M}}}$$

All established parameters used in the model can be found in Table 1.

**Table 1:** Parameter values and respective sources for all material constants used within COMSOL.

	Barium titanate	Cobalt ferrite
Electrical conductivity	10^–7^ S/m^111^	4.2 × 10^–5^ S/m^112^
Initial magnetic susceptibility	Not applicable	70^113^
Saturation magnetostriction	Not applicable	315 ppm^114^
Density	5700 kg/m^3^ (COMSOL)	6060 kg/m^3^ ^101^
Saturation magnetization	Not applicable	181,800 A/m ^115^
Poisson's ratio	Not applicable	0.33 ^116^
Relative permittivity	[1115.1, 1115.1, 1251.3] (COMSOL)	[9.0355, 9.0355, 10.5037] ^117^
Young's modulus	Not applicable	188.4 GPa ^118^
Elasticity matrix, voigt notation	[150.377, 656.308, 150.377, 65.9391, 65.9391, 145.521, 0, 0, 0, 43.8596, 0, 0, 0, 0, 43.8596, 0, 0, 0, 0, 0, 42.3729] [GPa] (COMSOL)	Not applicable
Coupling matrix, voigt notation	[[0, 0, − 4.32015, 0, 0, − 4.32015], [0, 0, 17.3624, 0, 11.4035, 0], [11.4035, 0, 0, 0, 0, 0]] [C/m^2^] (COMSOL)	Not applicable

Harmonics measurements of SPIONs and MENPs employed a time-dependent study with *f* = 25.25 kHz based on the M–H curve of CFO nanoparticles^101^ corresponding to the Langevin function and the negligible magnetic susceptibility of the BTO shell. The time step was 1 µs and the time range was 0–80 µs. The surrounding electrolyte medium was modeled as cerebrospinal fluid, and a cylindrical infinite element domain shell was placed outside the main rectangular region. The *Magnetic Field* boundary condition was applied to all faces immediately inside the infinite element domain, and electric fields were generated by the *Electric Potential* boundary condition applied to the top and bottom faces (Fig. S4). Rotation effects were modeled by fixing the applied electric field and rotating the applied magnetic field. Moreover, only coordinates within the particle were sampled for mean and decile plots, and a physics-controlled mesh with normal element size was used. Both stationary and time-dependent studies used a fully-coupled automatic highly nonlinear Newton node with an iterative linear FGMRES solver.

### Core size trend analysis

For core–shell CFO–BTO nanoparticles, the electric field and magnetic flux density plots for core radii ranging between 5 and 12 nm were modeled by changing the size of the inner semicircle (CFO) while maintaining the overall radius at 15 nm. 95% confidence intervals were defined as twice the standard error of either regression data or the regression slope. For the slope trend analysis, core sizes were simulated from 0 to 14 nm in increments of 1 nm. Core sizes larger than 14 nm had poor convergence due to numerical instability and were thus excluded from the analysis. Core size-dependent magnetization and magnetic flux density modulation slopes were derived using linear regression. The trend of these slopes with respect to core size was defined as the second derivative of magnetization (or magnetic flux density) and derived using quadratic curve fitting.

### Neuronal magnetization simulations

Nanoparticle magnetization changes were linearly mapped to electric field magnitude: a 0.02 A/m magnetization change per 50 mV/mm for a 12 nm core size was used, and the observed direction-dependent effect was applied. Electric field vectors were computed as the gradient of simulated extracellular voltage, and open-source Python libraries LFPy^119^ and NetPyNE^120^ were used for simulations of extracellular voltage around single neuronal morphologies and neural networks, respectively, integrated with the neural biophysics simulator NEURON^121^. MENP concentration was maintained at 117.5 µM (27.495 μg/mL), corresponding to 1415 particles/µm^3^.

### Neuronal morphologies

Biophysical parameters involving Allen Brain Atlas morphologies were obtained from previous studies^122^. The geometries were manually aligned to a three-dimensional template soma and simulated using Python LFPy^119^ and NEURON^121^. Human middle temporal gyrus layer 3, middle temporal gyrus layer 6, frontal lobe layer 3, and middle frontal gyrus layer 3 cells were simulated (n = 4 each). Action potentials were induced by raising the membrane potential, raising the sodium Nernst potential, and lowering the potassium Nernst potential. A 20 µm inclusion zone around subcellular compartments defined somatal, axonal, and dendritic voxel categories. Magnetization was quantified during the largest action potential peak within the first 20 ms, and the mean values for each cell compartment class were grouped in aggregate to define the significance between cell types. One-way ANOVA (*anova1*) in MATLAB R2021a (The MathWorks, Inc. Natick, MA, USA) was used to determine significance between cell type groups, with three different biophysical parameter sets yielding equivalent quantification significance outcomes.

### Monte Carlo simulations of realistic neural networks

Volumetric simulations were performed by reconstructing rat neocortical architecture^123^ using the Python library NetPyNE^120^. A 200 µm by 200 µm column of neocortex layers II, III, and IV was simulated, with depths of 150 µm, 350 µm, and 200 µm (total depth was 700 µm), and Excitatory:Inhibitory/Total ratios of 895:95/990, 988:188/1176, and 970:170/1140, respectively. Excitatory synapses were NMDA receptor-based (τ_1_ = 0.8 s, τ_2_ = 5.3 s, V_rest_ = 0 mV) and inhibitory synapses were GABA receptor-based (τ_1_ = 0.6 s, τ_2_ = 8.5 s, V_rest_ = − 75 mV). Excitatory cells were interconnected while inhibitory cells were only connected to excitatory cells. Excitatory synapses had a weight of $25\times {y}_{norm}$ mV (y_norm_ is the normalized neuronal depth) and a connection probability of $p$ = 0.1, while inhibitory synapses had a weight of 5 mV and a connection probability of ${p= 0.4e}^{-d/\lambda }$ (d is the synaptic distance; λ is the length constant of 150.0 µm^120,123^). The simulation time step was 100 μs and the extracellular voltage was recorded with a resolution of 5 µm and 1 ms. Nanoparticle concentration was maintained at 27.495 μg/mL. 40,000 particles were normally distributed within the x–z plane (200 µm × 200 µm), with the particle movement modeled by a diffusion coefficient^81^ D = 15 µm^2^/s and a perfusion velocity^79^ of 650 µm/ms.

## Supplementary Information

Supplementary Information.
