# Supplementary material for: In silico assessment of electrophysiological neuronal recordings mediated by magnetoelectric nanoparticles
Source: Sci Rep. 2022 May 19;12:8386. doi: 10.1038/s41598-022-12303-4 (PMC9120189; doi:10.1038/s41598-022-12303-4)
Supplement: Supplementary file 1 — Supplementary Information. [file 41598_2022_12303_MOESM1_ESM.pdf]

Supplemental Material

In Silico Assessment of Electrophysiological Neuronal Recordings Mediated by Magnetoelectric Nanoparticles

Ilhan Bok[a,b,e], Ido Haber[c], Xiaofei Qu[a], Aviad Hai[a,b,d,e*]

[a] Department of Biomedical Engineering, University of Wisconsin – Madison, Madison, WI, USA.

[b] Department of Electrical and Computer Engineering, University of Wisconsin – Madison, Madison, WI, USA.

[c] Department of Integrative Biology, University of Wisconsin – Madison, Madison, WI, USA.

[d] Grainger Institute for Engineering, University of Wisconsin – Madison, Madison, WI, USA.

[e] Wisconsin Institute for Translational Neuroengineering (WITNe), Madison, WI, USA.

Figure S1: Effect of MENP core size on internal strain. (a) The effect of core size on the ε_{33} component of strain (colormap). Core radius ranged from 5 to 12 nm corresponding to shell thicknesses ranging from 10 to 3 nm. In all cases, E_z = -100 mV/mm antiparallel to H_z = 4 kA/m.

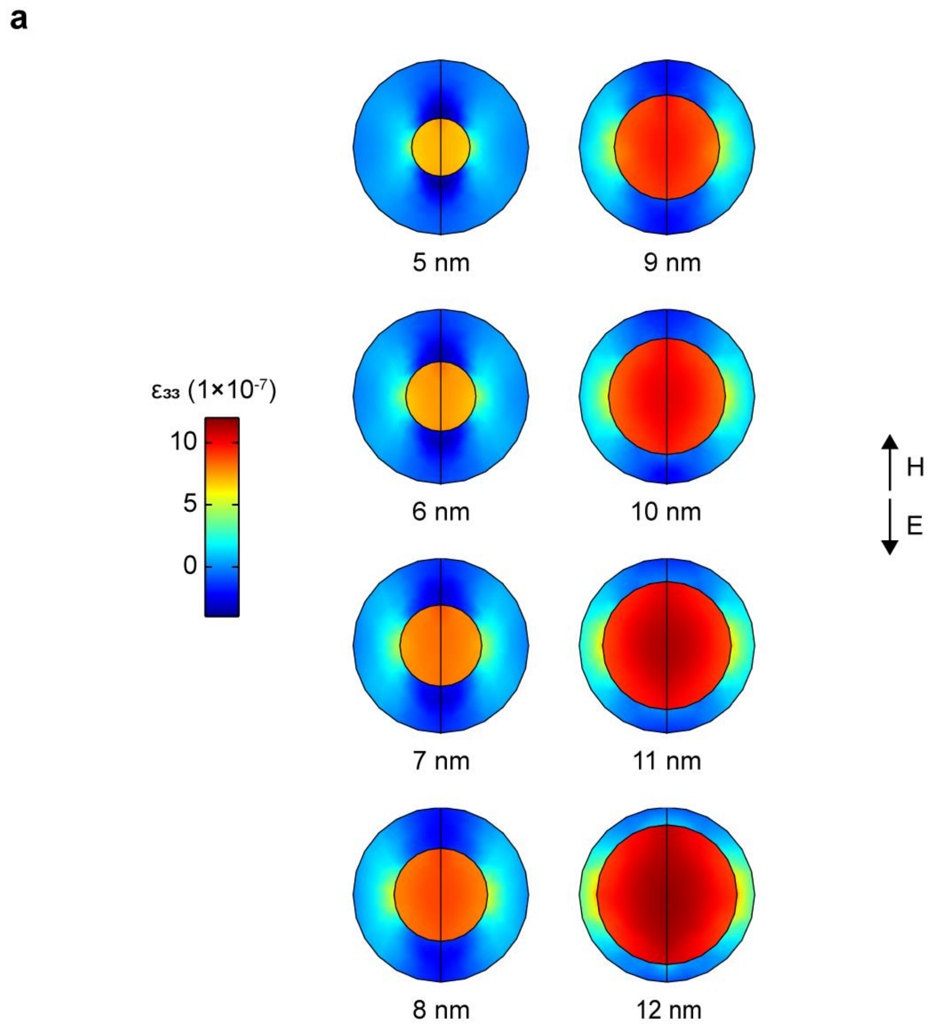

Figure S2: Nanoparticle strain and displacement at high fields comparable to extant literature on magnetoelectric composites. (a) The 33 component of strain (unitless, 10^{-4}) and (b) displacement (nanometers, 10^{-2}) at E_z = -10 kV/cm antiparallel to Hz = 1 kOe (approximately 80 kA/m). Disp. = displacement

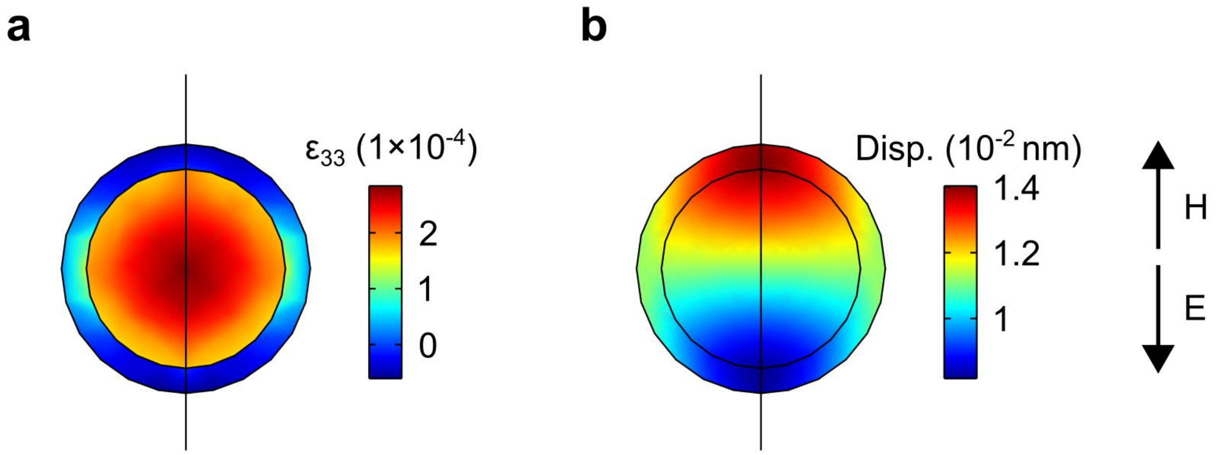

Figure S3: Effect on magnetization of electric field and magnetic field intensity relative direction. (a) Magnetization norm plots for {0°, 30°, 45°, 60°, 90°, 120°, 135°, 150°} magnetic field rotation directions. (b) Magnetization average z-component plot versus application angle. (c) Magnetization decile plot; deciles plotted are (0-100), (10-90), (20-80), (30-70), (40-60), and the mean (50th percentile).

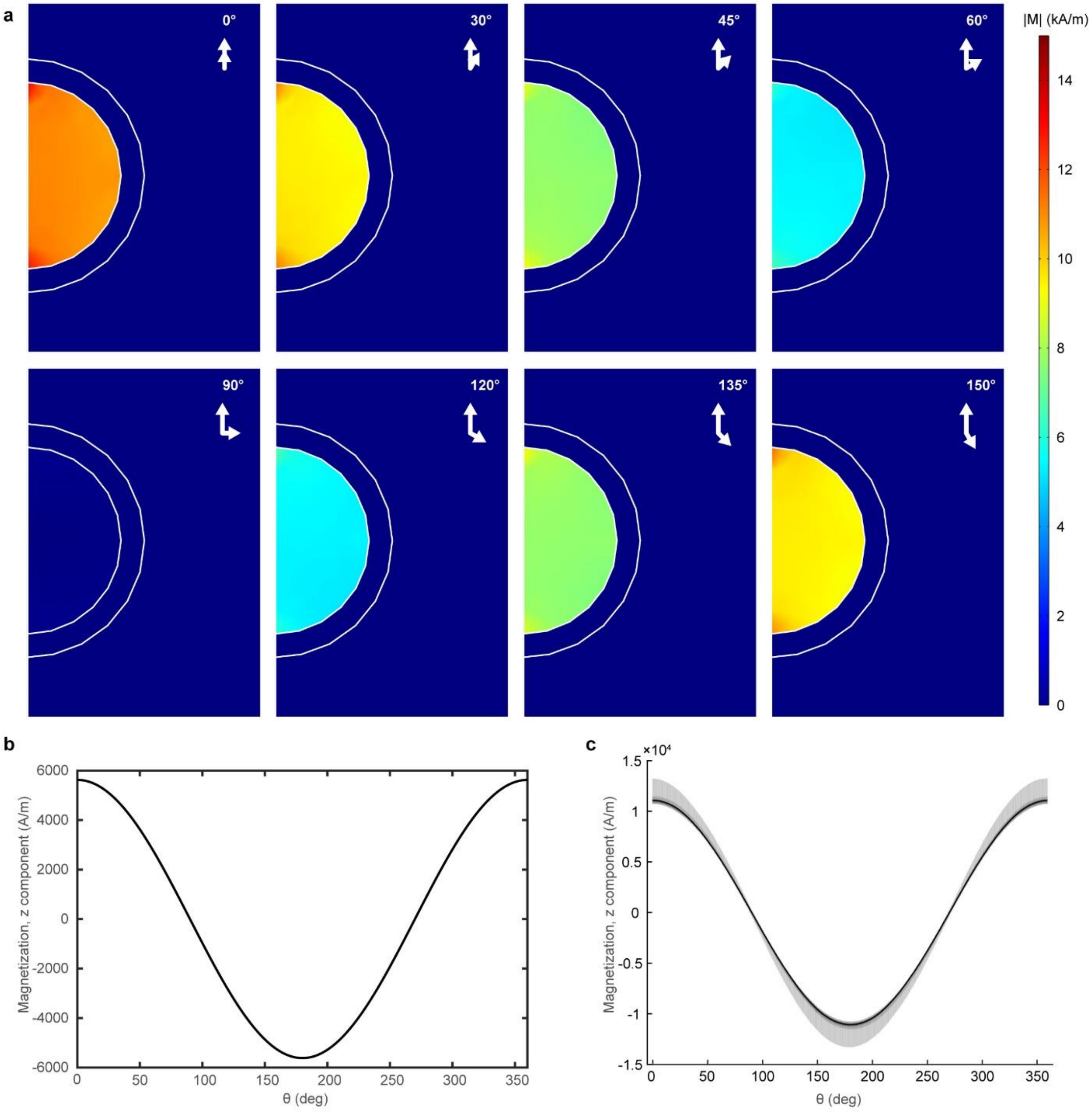

Figure S4: Boundary/domain conditions and mesh placement in COMSOL Multiphysics. (a) Grounding the bottom boundary of the domain and applying voltage to the top boundary allows for electric field creation (red.) Application of a magnetic field intensity boundary condition (green.) Infinite element domain application to the outer shell (blue.) (b) Model meshing arrangement used ("normal" density).

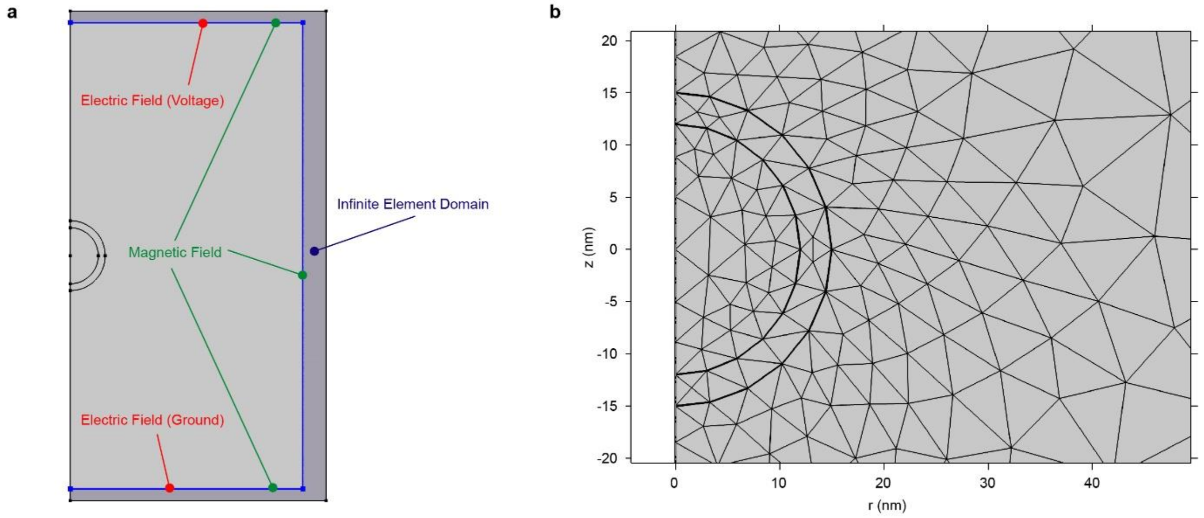

Table S1: Detailed statistical parameters of ANOVA analysis of *in silico* neuronal morphologies

Compartment (Type)	F	n (total)	p
Aggregate	24.5	4	7.90429×10^{-16}
(MTG3)		10452	
(MTG6)		3399	
(FLL3)		8505	
(MFG3)		11291	
Soma	1.73	4	0.21473
(MTG3)		4	
(MTG6)		4	
(FLL3)		4	
(MFG3)		4	
Axon	1.06	4	0.40931
(MTG3)		3	
(MTG6)		4	
(FLL3)		3	
(MFG3)		4	
Dendrite	5.11	4	0.01655
(MTG3)		4	
(MTG6)		4	
(FLL3)		4	
(MFG3)		4	
